# The patterns of *Corylus* and *Alnus* pollen seasons and pollination periods in two Polish cities located in different climatic regions

**DOI:** 10.1007/s10453-013-9299-x

**Published:** 2013-03-14

**Authors:** Małgorzata Puc, Idalia Kasprzyk

**Affiliations:** 1 Department of Botany and Nature Conservation, University of Szczecin, Felczaka 3c, 71-412 Szczecin, Poland; 2 Department of Environmental Biology, University of Rzeszów, Zelwerowicza 4, 35-601 Rzeszów, Poland

**Keywords:** *Corylus*, *Alnus*, Phenology, Pollination, Pollen season, Poland

## Abstract

This study compares phenological observations of *Corylus* (hazel) and *Alnus* (alder) flowering with airborne pollen counts of these taxa recorded using volumetric spore traps (2009–2011). The work was carried out in the Polish cities of Szczecin and Rzeszów that are located in different climatic regions. Correlations between pollen concentrations and meteorological data were investigated using Spearman’s rank correlation analysis. The timings of hazel and alder pollination and the occurrence of airborne pollen varied greatly and were significantly influenced by meteorological conditions (*p* < 0.05). The flowering synchronization of hazel and alder pollination in Szczecin and Rzeszów varied over the study period. Hazel and alder trees flowered notably earlier in stands located in places that were exposed to sunlight (insolated) and sheltered from the wind. On the other hand, a delay in the timing of pollination was observed in quite sunny but very windy sites. In Rzeszów, maximum hazel pollen concentrations did not coincide with the period of full pollination (defined as between 25 % hazel and alder and 75 % of flowers open). Conversely, in Szczecin, the highest hazel pollen concentrations were recorded during phenophases of the full pollination period. The period when the highest alder pollen concentrations were recorded varied between sites, with Rzeszów recording the highest concentrations at the beginning of pollination and Szczecin recording alder pollen throughout the full pollination period. Substantial amounts of hazel and alder pollen grains were recorded in the air of Rzeszów (but not Szczecin) before the onset of the respective pollen seasons.

## Introduction

The presence of pollen in the air at a particular place is the result of interactions between factors that affect the formation of inflorescences, pollination, and the transport of pollen in the atmosphere. The proportion of a pollen-producing taxon in the vegetation of a particular region and the response of this taxon to weather conditions are the most important factors determining temporal variations in pollen concentrations in the atmosphere (Aboulaïch et al. 2008; Guardia and Belmonte 2004; Jato et al. 2007a, b; Sugita et al. 2010). Knowledge of these relationships helps the interpretation of aerobiological data and allows the construction of more accurate models for predicting concentrations of allergenic pollen in the air.

Hazel (*Corylus* L.) and alder (*Alnus* Mill.) trees are common throughout Poland. Both genera belong to the Fagales Engl. order and the Betulaceae S. F. Gray family, which also includes *Betula* and *Carpinus* (APG II 2003). In Europe, the *Corylus* pollen type includes three species that are morphologically similar (*Corylus avellana* L., *C.* *colurn*a L., and *C.* *maxima* Mill.). Hazel is absent from the northernmost parts of Europe and from some coastal regions of southern Europe, particularly the Iberian and Balkan Peninsulas. *C. avellana* is the most widespread species of hazel and is present across almost the whole of Europe, occurring as far south as the Caucasus Mountains and the Crimean Peninsula. *C. avellana* occurs in natural habitats throughout Poland; it is common in forests and clearings, including the lower montane forest zone. *C. avellana* prefers sunny sites, temporarily or periodically shaded and grows on a wide variety of soils: dry to moist and fertile; humid-mineral to sandy clay; stony clay. It is a very tolerant species with regard to light conditions and can grow well even in shaded sites. It plays an important role in biocoenosis—it provides shaded conditions for soil formation, to which it contributes easily decomposing litter; it also supplies nuts for rodents and birds to consume (Senata 1991).

The occurrence of *C. colurna* and *C. maxima* is restricted to certain areas of south-eastern Europe (Jalas and Suominen 1988; Bugała 2000). However, *Corylus colurna* (Turkish hazel) is widely cultivated as an ornamental tree in Europe and is seen in many cites of Poland. It is very tolerant of difficult growing conditions in urban situations, which has increased its popularity in urban planting schemes in recent decades (Stachak et al. 2000).

Four species of alder occur in Europe: (1) *Alnus glutinosa* (L.) Gaerther; (2) *A. incana* (L.) Moench.; (3) *A. viridis* (Chaix) DC. in Lam. & DC.; and (4) *A. cordata* (Loisel.) Loisel. The latter occurs only in Corsica and in south-western Italy (Huntley and Birks 1983). In Poland, the genus *Alnus* Mill. is represented by three species. Two are trees, *A. glutinosa* and *A. incana*, and the third (*A. viridis*) is a small shrub confined to the Bieszczady Mountains. The ranges of each of the three species of alder in Poland are different. Apart from quite important geographical variations, there are also some ecological differences. *A. glutinosa* is common throughout the country, but it avoids higher altitudes in the mountains. *A. incana* is less common; it is mainly situated in southern Poland and along the course of the Vistula River (Zając and Zając 2001). Both species occur on mineral and organic soils, but the natural and semi-natural tree-stands of *A. glutinosa* grow on peaty soils. On the other hand, *A. incana* demands mainly young alluvial soils. They display a similar range of soil requirements in regard to acidity and moisture, although *A. incana* is able to tolerate lower moisture conditions. Both species are characterised by a tolerance of considerable fluctuations of water level. *A. glutinosa* and *A. incana* can be regarded as pioneer plants easily occupying new or previously disturb habitats. If grown in the same habitat, *A. incana* flowers several days to 3 weeks prior to *A. glutinosa* (Pancer-Kotejowa and Zarzycki 1980).

In Poland, in addition to hazel pollen, alder pollen is considered to be the most important cause of airborne allergy diagnosed at the beginning of the growing season. Pollen concentrations are high, and they often exceed the threshold values causing allergy symptoms (Rapiejko et al. 2004; Puc 2007; Weryszko-Chmielewska and Rapiejko 2007; Kaszewski et al. 2008). The high degree of cross-reactivity between the major allergens in hazel and the major allergens in other members of the Betulaceae family (i.e. alder and birch) means that it is important to monitor this pollen type even though atmospheric concentrations of hazel pollen are usually considerably lower (Puc 2003a, b; D’Amato et al. 2007).

In Poland, hazel and alder are the first pollen grains to appear in the air. The onset of pollen seasons and the overall curve of the pollen season largely depend on meteorological conditions before and during pollen release. Hazel and alder set male inflorescences in late summer in the year preceding pollination and a period of dormancy, called the chilling period, are required to enter a new growing cycle. After the dormancy period, the plant is ready to begin growth processes, but this coincides in time with a period of low or subzero temperatures that are adverse to growth. The forced dormancy period lasts until the day when the cumulative temperature reaches a species-specific threshold temperature (thermal energy) (Suszka 1980). For the species in question, large variations are observed in both pollination timing and the start of the pollen season (Kozłowski 1971; Cenci et al. 1997; Jato et al. 2004; Rodriguez-Rajo et al. 2004; Črepinšek et al. 2006; Puc 2007; Kaszewski et al. 2008; Hájková et al. 2009). It has been shown in Poland that in the 1950s, flowering isophenes of hazel female flowers closely corresponded to the isotherms of March. Currently, hazel blooms earlier; hence, the temperatures in January/February are of major importance for the initiation of flowering (Sokołowska 1962; Kasprzyk 2011). Weather patterns significantly affect daily hazel and alder pollen concentrations. The most important factors include temperature, precipitation, humidity, sunlight hours, and wind speed. The strength of correlations between these meteorological elements and pollen counts is different during the pre-peak and post-peak periods of the pollen season (Rodriguez-Rajo et al. 2004; Puc 2007).

Meteorological factors have a strong impact on variations in the production, release, and dispersal of allergenic pollen. As a result, these variables can be used for constructing forecast models (Puc 2012). Recent attempts have been made to include pollination (phenological observations) as a biotic factor in such models, and research has included comparing variations in pollination and the occurrence of airborne pollen (Latorre 1997; Kasprzyk and Walanus 2007; Jato et al. 2004; Stach et al. 2006; Jato et al. 2007a, b).

The overall climate of a region affects the seasonality of natural phenomena, hence also the pollination and the occurrence of airborne pollen. Therefore, the present study was carried out at sites located in different climatic regions. A hypothesis was, therefore, put forward that the curve of the pollen seasons and the timing of hazel and alder pollination would differ in these regions. The main objectives were to determine whether the hazel and alder pollen seasons coincided with the pollination period, and whether the relationships between these phenomena were similar in the two cities studied.

## Materials and methods

### Site location

**Fig. 1:**
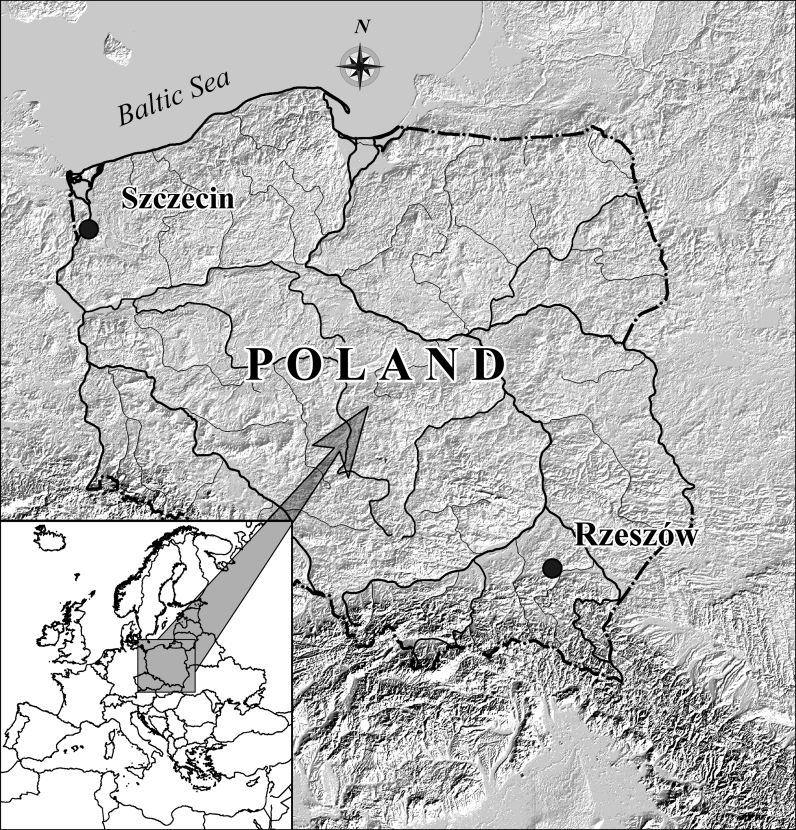
The geographic location of Szczecin and Rzeszów in central Europe

Aerobiological monitoring (2009–2011) was carried out in two cities, Szczecin (53°26′26″ N, 14°32′50″ E) and Rzeszów (50°01′N; 22°02′E). The cities are located 640 km apart and situated in different climatic regions (Fig. 1); the westerly circulation from the North Atlantic has varying degrees of influence on the climate of the two cities (Ziernicka-Wojtaszek and Zawora 2008).

Szczecin is the capital of Western Pomerania, and it is situated in north-western Poland. The area immediately around the city consists of forested land (there are three forests near to the city) as well as some hills and water bodies. Within the city area, there are introduced synanthropic plants and trees as well as primeval forests. Forests occupy more than 16 % of the Szczecin County area. The green areas of the city also include numerous parks, lawns, and gardens. As a result of spatial development of the city, several types of residential districts have appeared. The city centre is occupied by tall buildings with rented flats, characteristic of the late nineteenth and early twentieth century. Only the northern part of the city centre is dominated by old villas. The majority of the city’s districts are covered with villas or low houses and also blocks of flats (Koźmińska and Wojciechowska 2001).

The centre of Rzeszów lies in the valley of the Wisłok River, 200–215 m above sea level. In this region, there are no natural barriers and the altitude ranges between 80 and 200 m a.s.l. The city is more than a dozen kilometers from the Carpathian Foothills (300–600 m a.s.l.). Rzeszów is a medium-sized city with typical urban developments. Its vegetation is concentrated in parks and urban lawns (Święs 1993). Near the pollen trap, there are several planted trees and shrubs as well as synanthropic plant associations. The environs of Rzeszów are a mosaic of forests and crop fields; agricultural land accounts for a major part in the land use structure. Forests occupy about 20 % of Rzeszów County area.

### Climate

The climate of Szczecin is modified by the influence of Atlantic air masses and the proximity of the Baltic Sea. It has humid continental climate, with January the coldest month (−1.1 °C) and July the hottest (17.7 °C). The average annual temperature is 8.4 °C, annual mean relative humidity ranges between 70 and 77 %, and rainfall is mainly concentrated in summer. Mean annual precipitation is 528 mm. Daily precipitation of over 20 mm or draught periods lasting longer than 20 days are rare. The climate of Szczecin includes strong and very strong winds that are especially frequent from November till March. The average monthly wind speed over the years 1956–1990 is 3.6 m/s (Koźmiński and Czarnecka 1996; Woś 1999). The vegetation season, which is the period with mean 24-h air temperature >5 °C and in Poland in the moderate climate zone lasts from the last spring ground frost to the first autumn ground frost, is about 210–220 days in Szczecin (Kozuchowski and Degirmendžic 2005).

Rzeszów is located in south-eastern Poland. Its climatic conditions are chiefly affected by transformed polar maritime and polar continental air masses. During the year, there are roughly 230 days with transformed maritime air masses (Niedźwiedź 1981, 2004). The city is situated in a region where very warm days with precipitation occur frequently in summer, and days with ground frost conditions and cool or very cool sunny weather occur in winter. There are few cool days with precipitation and high cloudiness (Woś 1999). In this region, western, north-western, and south-western winds predominate. Mean wind velocity for Rzeszów is 4 m/s. The average annual air temperature oscillates around 8 °C in the Carpathian Foothills, which makes it one of the warmest regions in Poland. The active growing season lasts from 215 to 220 days in Carpathian Foothills (Niedźwiedź 2004). The mean annual temperature is 8.1 °C, and mean annual precipitation is 633 mm. Mean temperatures for July (the warmest month) and January (the coldest month) are 18.3 and −2.1 °C, respectively. In the Rzeszów region, maximum precipitation occurs in July (mean 80 mm) and minimum precipitation in February (mean 27–50 mm) (Brzeźniak 2007).

### Aerobiological monitoring (pollen counts)

In both towns, aerobiological monitoring was conducted using volumetric spore traps of the Hirst design (Hirst 1952). In Szczecin, the pollen trap was set at a height of about 21 m above ground level, whereas in Rzeszów, it was at 12 m above ground level. Two different counting methods were employed in this study (these two methods of slides’ counting are consistent with International Association for Aerobiology (IAA) recommendation). In Szczecin, slides were examined along 4 longitudinal transects divided into 2-mm intervals. In Rzeszów, pollen grains were counted along 12 transversal transects, each corresponding to a 2-h interval. Pollen grains were identified and counted using light microscope at magnification 400×. The results were expressed as the daily average number of pollen grains in 1 m^3^ of air per 24 h.

The pollen season was defined using the 95 % method; the day on which the cumulative pollen count during the period 1 January–30 June reached the value of ≥2.5 % was determined to be the start date of the pollen season, and the end of the season was the day when the cumulative pollen count was ≥97.5 % (Nilsson and Persson 1981; Jato et al. 2006). The total pollen count over this period was expressed by the symbol SPI (Seasonal Pollen Index). The distributions of the data were not normal (Shapiro–Wilk test); therefore, Spearman’s rank correlation analysis (StatSoft Inc 2008) was used to evaluate the synchronization of the pollen seasons in these two cities and to analyse the correlations between pollen concentrations and weather conditions. In the Spearman’s rank analysis, the following factors were selected as a group of independent variables: daily maximum temperature (Tmax), daily minimum temperature (Tmin), daily average temperature (Tmean), PP (precipitation), H % (relative humidity) on the previous days (*n* − 2, *n* − 1) and on the current day. The analysis of data from the previous day was taken into account because pollen release is not only affected by the weather conditions in the current day, but also those in 1–2 days preceding pollination (Uruska 2003). In these statistical tests, the level of significance was set at *p* < 0.05.

### Pollination (phenological observations)

**Fig. 2:**
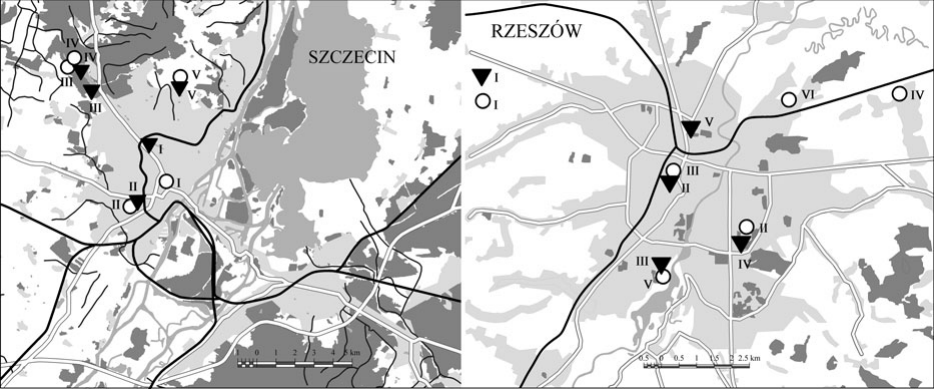
The sites observations of *Corylus* and *Alnus* in Szczecin and Rzeszów (*triangles* sites of *Corylus*, *circles* sites of *Alnus*)

Phenological observations were carried out each year from the 1st of January until the end of flowering of the last of the investigated species (Fig. 2). The Łukasiewicz method (1984) was used to record the successive phenophases (F) of the generative development of plants:

1. The appearance of the first flowers or inflorescence buds; hazel and alder set inflorescence buds in late summer of the previous year, so phase F1 was counted from the 1st of January and so the first phenological observations started from phase F2;
2. Blooming of the first flowers;
3. The beginning of full flowering (25 % of flowers open);
4. The first flowers being shed (withered);
5. The end of full flowering (75 % of flowers open);
6. The last flower buds;
7. The end of flowering (from the day when the last flowers ended blooming until the end of June; the end of June was chosen arbitrarily). The full pollination period includes phases from F3 to F5.

**Fig. 3:**
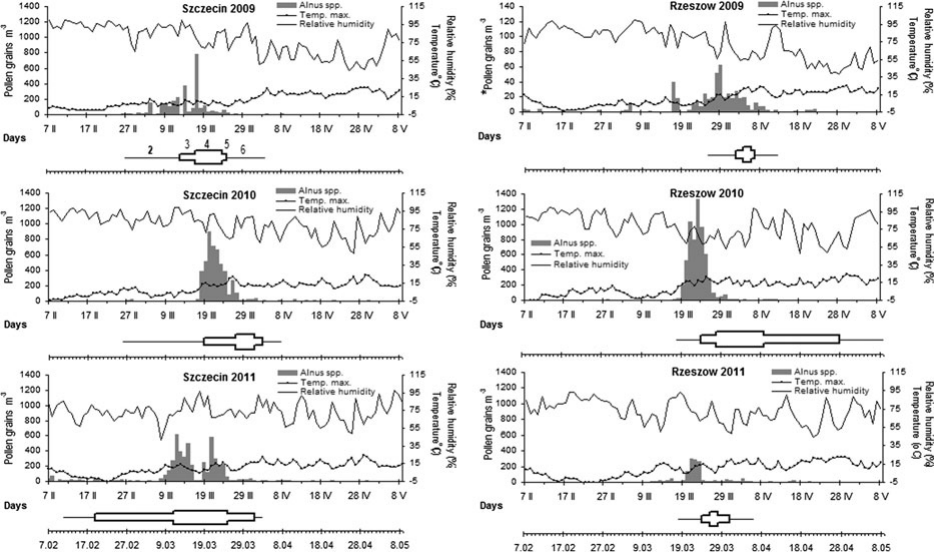
The *Alnus* pollen count versus meteorological conditions and average terms of phenophases: 2—F2, 3—F3, 4—F4, 5—F5, 6—F6; in Szczecin and Rzeszów (2009–2011)

**Fig. 4:**
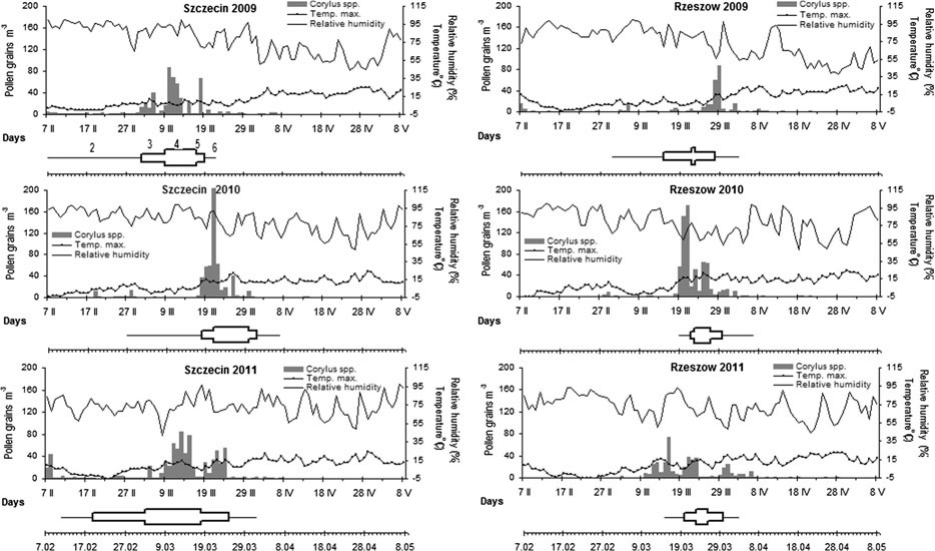
The *Corylus* pollen count versus meteorological conditions and average terms of phenophases: 2—F2, 3—F3, 4—F4, 5—F5, 6—F6; in Szczecin and Rzeszów (2009–2011)

The observations were conducted at intervals of a few days, depending on the rate of flowering. During the full pollination period (F3–F5), observations were made more frequently, that is, every 2 to 3 days at five or six sites established for the two species. The observations were carried out on 3 individuals growing at the same sites and, where possible, over a period of 3 years. If it was not possible to carry out observations at these same sites (e.g. because a particular tree/shrub had been cut down), some individuals growing closest to this place and under similar habitat conditions were observed. The habitat conditions at the same site were identical, so on the basis of observations, the average duration of the successive phenological phases was determined. This permitted accurate reference of the phenophases to the pollination seasons (see Figs. 3, 4). *C. avellana* and *A. glutinosa* occur in both cities and in their surroundings:

- Szczecin—No *Corylus* trees but 3 *A. glutinosa* individuals were found within about 200 m of the pollen sampling site. About 140 *Corylus* trees and more than 150 *A. glutinosa* trees and shrubs, but only one *A. incana* individual, were found within a 5 km radius from the pollen trap.
- Rzeszów—Four (4) *Corylus* individuals were found, but there were no *A. glutinosa* or *A. incana* at a distance of up to 100 m. The closest site with *A. glutinosa* was about 500 m from the pollen trap. No *A. incana* stands were found within a 5 km radius from the pollen trap.

By comparing the timing of flowering periods at the chosen sites, the synchronization index (X) was calculated according to the formula proposed by Ollerton and Lack (1998), see Table 4.

$$ x_{1} = \left( {\frac{1}{n - 1}} \right)\left( {\frac{1}{{f_{1} }}} \right)\sum\limits_{j = 1}^{n} {e_{j} \ne i} $$

*e*_*j*≠*i*_—the number of days individuals *i* and *j* overlap in their flowering, *n—*number of individuals on each site, *f*_*i*_—total number of days of flowering.

An index close to 1 indicates a high level of flowering synchronization.

*Corylus avellana* and *Alnus glutinosa* (designated as: I, II, III, IV, V, VI—Table 4) lying within a 5 km radius of the city and characterised by varied environmental conditions such as exposed to sunlight (insolated), shade, soil moisture content, and wind: The site descriptions are given below:

#### Szczecin

*Corylus avellana*: (I) insolated, close to a watercourse, shielded against wind, in a park in the city centre; (II) partly shaded, in a shallow land depression, in a park, close to a watercourse; (III) a shaded place, on a lake escarpment in the forest; (IV) partly shaded, at the forest edge, not shielded against the wind; (V) insolated, in a shallow land depression, not shielded against wind, in winter extended period of snow cover.

*Alnus glutinosa*: (I) moderately insolated, partly shielded by buildings; (II) partly shaded, in a small land depression, in a park, close to a watercourse, wind shielded; (III and IV) on a lake bank, in spring periodically flooded, not shielded against wind; V—in a small land depression, close to a watercourse, poorly insolated.

#### Rzeszów

*Corylus avellana*: (I) insolated, near terraced houses; (II) in a city park, a shaded place close to tall trees; (III) in undergrowth of a deciduous forest, near the bank of the Wisłok River; (IV) shielded against wind, insolated, in an area with detached houses; (V) insolated, close to a block housing estate.

*Alnus glutinosa*: (I) insolated and not shielded against wind; (II) at the Wisłok River bank, not shielded against wind; (III) in the city centre, near a railway track, partly shaded; (IV) shaded, on a street, close to a block housing estate; (V) insolated, periodically flooded in spring and autumn; (VI) near a drainage ditch, close to allotment gardens, shielded against wind (Fig. 2).

### Pollen seasons and pollination periods

For each phenophase, the pollen sum was calculated and then expressed as a proportion. The similarity in the distribution of grain numbers was tested by *χ* ^2^ at *p* < 0.05.

## Results

### Characteristics of the pollen seasons

#### General characteristics

Start dates of the pollen seasons of the studied taxa varied greatly. In Szczecin, over the 3-year study period, the differences were 42 days for hazel and 38 days for alder. In Rzeszów, the differences were lower; 33 days for hazel and also 33 days for alder. The end dates of pollen seasons were characterised by much smaller variations. In both Szczecin and Rzeszów, the maximum hazel and alder pollen concentrations generally occurred in the second half of March.

The pollen seasons varied considerably between the years and the monitoring stations. Generally, the first hazel and alder pollen grains appeared earlier in Szczecin compared with Rzeszów.

In 2009, concentrations of hazel and alder pollen grains recorded in the air of Szczecin increased rapidly while the increase was much slower in Rzeszów. In Szczecin, alder pollen remained in the air longer than hazel pollen. In 2010, the dynamics of the *Alnus* pollen seasons were similar for both cities, in particular in the pre-peak period, that is, until the peak date. In 2011, two peaks were noted in Szczecin for both taxa. For *Corylus* and *Alnus* in Szczecin, the start date was the earliest over the 3-year study period. In Rzeszów, the lowest values of pollen count were observed for *Corylus* and *Alnus* (Figs. 3, 4).

#### Skewness and Kurtosis

*Skewness* The pollen seasons were generally strongly skewed to the right (Table 1), which means that single pollen grains of both hazel and alder remained in the air for a long time after the pollen season had ended. Single grains of *Alnus* were even observed into June in Szczecin (in 2010) and the end of May in Rzeszów.

**Table 1:** Descriptive statistics of *Corylus* and *Alnus* pollen seasons in Szczecin (SZ) and Rzeszów (RZ) (2009–2011)

	Start	End	Length/days	SPI	Maximum	Date of maximum	Skewness	Kurtosis
*Corylus*								
SZ 2009	2 III	27 III	26	472	86	9 III	1.62	1.58
SZ 2010	20 III	30 III	34	528	205	21 III	3.13	11.34
SZ 2011	7 II	28 III	50	783	85	13 III	1.87	2.75
RZ 2009	7 II	8 IV	62	297	88	29 III	4.99	26.87
RZ 2010	12 III	2 IV	21	653	170	21 III	2.16	4.23
RZ 2011	10 III	28 III	19	337	73	16 III	2.08	5.59
*Alnus*								
SZ 2009	2 III	4 IV	34	2698	778	17 III	3.66	15.76
SZ 2010	18 III	8 IV	23	4566	898	20 III	1.18	2.20
SZ 2011	9 II	31 III	51	4728	619	12 III	2.05	3.58
RZ 2009	15 II	12 IV	69	454	63	29 III	2.35	6.24
RZ 2010	19 III	30 III	11	6001	1325	23 III	0.59	1.00
RZ 2011	13 III	23 IV	41	1460	295	2 III	3.01	8.12

*SPI* seasonal pollen index

*Kurtosis* All kurtosis values were positive, which showed that pollen counts usually increased rapidly at the beginning of the season. For alder, the values of the kurtosis were usually higher in Szczecin than in Rzeszów.

When analysed according to each feature (i.e. year, city, and taxon), the seasonal curves were skewed to the right and strongly peaked, that is to say with narrow full-width half-maximum (FWHM). This is evident by the very high coefficients of skewness and kurtosis.

### The effect of weather on daily pollen counts

The effect of the main meteorological parameters was showed in Table 2. The variables Tmean, Tmin, and Tmax had an effect on daily average hazel and alder pollen grains recorded in 2009 in both cities. Relative humidity also had a negative effect on *Alnus* pollen counts.

**Table 2:** *Corylus* and *Alnus* pollen counts (95 % of the total pollen sum) and selected meteorological variables in Spearman’s rank correlation analysis for Szczecin and Rzeszów (2009–2011) with the current weather (*n*), the previous day’s weather (*n* − 1), and the weather 2 days earlier (*n* − 2)

Taxa	City	*T*_max_	*T*_min_	*T*_mean_	Precipitation	Humidity
2009						
*Corylus* spp.	Rzeszów (*n*)	0.4333	0.40119	0.4673	NS	NS
	Szczcin (*n*)	NS	NS	NS	NS	NS
	Rzeszów (*n* − 1)	0.4109	NS	0.3478	NS	−0.4029
	Szczcin (*n* − 1)	NS	NS	NS	NS	−0.4916
	Rzeszów (*n* − 2)	0.3612	NS	0.2624	NS	−0.3181
	Szczcin (*n* − 2)	NS	0.3316	NS	NS	NS
*Alnus* spp.	Rzeszów (*n*)	0.5972	0.5284	0.6041	NS	−0.5228
	Szczcin (*n*)	NS	NS	−0.2254	NS	−0.4417
	Rzeszów (*n* − 1)	0.5595	0.4383	0.5410	NS	−0.3709
	Szczcin (*n* − 1)	0.3922	NS	NS	NS	−0.4211
	Rzeszów (*n* − 2)	0.5351	0.3109	0.4609	NS	−0.4553
	Szczcin (*n* − 2)	NS	NS	−0.2327	NS	NS
2010						
*Corylus* spp.	Rzeszów (*n*)	0.7102	0.6409	0.7157	−0.6481	−0.6152
	Szczcin (*n*)	0.5633	NS	0.5917	NS	NS
	Rzeszów (*n* − 1)	0.5151	0.4960	0.5087	−0.6507	−0.6490
	Szczcin (*n* − 1)	NS	0.4498	0.5352	NS	NS
	Rzeszów (*n* − 2)	NS	NS	NS	−0.6293	NS
	Szczcin (*n* − 2)	NS	NS	NS	NS	NS
*Alnus* spp.	Rzeszów (*n*)	NS	NS	NS	NS	NS
	Szczcin (*n*)	NS	NS	NS	NS	NS
	Rzeszów (*n* − 1)	NS	NS	NS	NS	NS
	Szczcin (*n* − 1)	NS	NS	NS	NS	NS
	Rzeszów (*n* − 2)	NS	NS	NS	NS	NS
	Szczcin (*n* − 2)	NS	NS	NS	NS	NS
2011						
*Corylus* spp	Rzeszów (*n*)	NS	NS	NS	NS	NS
	Szczcin (*n*)	0.7187	0.5247	0.6375	NS	NS
	Rzeszów (*n* − 1)	NS	NS	NS	NS	NS
	Szczcin (*n* − 1)	0.5941	0.4464	0.5525	NS	NS
	Rzeszów (*n* − 2)	NS	NS	NS	NS	NS
	Szczcin (*n* − 2)	0.5246	0.2875	0.4354	NS	NS
*Alnus* spp.	Rzeszów (*n*)	−0.3731	NS	−0.3983	−0.3910	NS
	Szczcin (*n*)	0.7180	0.6390	0.7164	NS	NS
	Rzeszów (*n* − 1)	NS	NS	NS	−0.3477	NS
	Szczcin (*n* − 1)	0.6081	0.5491	0.6373	NS	NS
	Rzeszów (*n* − 2)	NS	NS	NS	NS	NS
	Szczcin (*n* − 2)	0.5571	0.4881	0.5628	NS	NS

*NS* statistical significance *α* ≤ 0.05

In 2010, in both cities, daily weather conditions during the season had a large influence on daily average hazel pollen counts but no impact on the amount of alder pollen in the air. Tmax and Tmean recorded on the previous day and current day had a positive effect on daily average pollen counts of *Corylus* in both cities. In Rzeszów, the number of airborne hazel pollen grains was found to be negatively affected by precipitation and humidity of the current and previous day.

In 2011, airborne concentrations of *Corylus* and *Alnus* correlated with the temperature of the current day, previous day, and 2 days earlier only in Szczecin. In Rzeszow, pollen count of *Corylus* did not correlate with the temperature. For *Alnus,* the negative correlation of pollen count is incorrect. However, the daily average hazel pollen counts did not depend on precipitation and humidity. Humidity did not have a statistically significant influence on daily average alder pollen concentrations in the two cities in 2011.

Rainfall has no effect at all on the change in the number of airborne pollen grains over the 3-year study period.

### Characteristics of pollination periods

No clear relationships were found in the timing of the full pollination period between the cities studied (Table 3).

**Table 3:** Descriptive statistics of the pollination periods in Rzeszów (RZ) and Szczecin (SZ) (2009–2011)

Years	Start (F2–F7)	End (F2–F7)	Length (F2–F7)	Start of (F3–F5)	End (F3–F5)	Length (F3–F5)
*Corylus*						
2009 SZ	7 II	21 III	43	03 III	18 III	16
2010 SZ	27 II	06 IV	39	18 III	31 III	14
2011 SZ	11 III	31 III	49	19 II	24 III	34
2009 RZ	02 III	02 IV	32	15 III	27 III	13
2010 RZ	19 III	06 IV	19	22 III	29 III	8
2011 RZ	15 III	02 IV	19	20 III	29 III	10
*Alnus*						
2009 SZ	27 II	06 IV	39	13 III	24 III	12
2010 SZ	26 II	07 IV	41	19 III	02 IV	15
2011 SZ	11 II	02 IV	51	19 II	31 III	41
2009 RZ	26 III	12 IV	18	02 IV	06 IV	5
2010 RZ	18 III	08 V	52	24 III	27 IV	35
2011 RZ	18 III	05 IV	19	24 III	30 III	7

#### Start of pollination periods (F2)

In Szczecin, both hazel and alder started to shed pollen much earlier than Rzeszów. For instance, alder occurred as much as 28 days earlier in Szczecin 2009. The onset of pollen shed in Szczecin always occurred in February, whereas in Rzeszów it occurred in March. For hazel, the average start dates were the 15th of February for Szczecin and the 12th of March for Rzeszów (Table 3; Figs. 3, 4). On average, alder started to pollinate on the 19th of February in Szczecin and on the 20th of March in Rzeszów.

#### End of pollination periods (F7)

The end dates of the pollination period were usually similar. There was one exception in the year 2010 when the pollination period only of *Alnus* ended much earlier in Szczecin compared with Rzeszów; in Rzeszów, the end of flowering was recorded a month later at the beginning of May. On average, the end of the pollination period of hazel occurred on the 5th of April and alder pollination ended on the 30th of March in Szczecin, whereas in Rzeszów, pollination ended on the 3rd of April and the 18th of April for hazel and alder, respectively.

#### Duration of the full pollination periods (F3–F5)

The duration of the full pollination period clearly differed between the cities. The full pollination period of *Corylus* ranged from 14 to 34 days at Szczecin (mean 21 days) and in Rzeszów the period F3–F5 ranged from 8 to 13 days (mean 10 days). For *Alnus,* the full pollination period was between 12 and 41 days at Szczecin (mean 23 days) and 5–35 days at Rzeszów (mean 16 days) (Table 3; Figs. 3, 4).

Clear differences in the duration of the particular phenophases of the two species studied were also noted between Szczecin and Rzeszów. The phenological phases F2 and F4 for hazel and alder were usually longer in Szczecin than in Rzeszów. An exception occurred in 2010 for phase F4 of alder, which was shorter in Szczecin than in Rzeszów (Table 3; Figs. 3, 4).

#### Synchronization of pollination within the two cities

In Szczecin, the synchronization of pollination in the study period varied (Table 4). The highest synchronization index was found in 2011, and the mean was very similar for both taxa. On the other hand, clear differences in pollination timing were observed in 2009 and 2010. In these years, both *Corylus* and *Alnus* bloomed earliest, in the stand located in a sunny place and sheltered from the wind. The lowest synchronization index was recorded for *Corylus* at site II in 2010, and this was because over 50 % catkins in two out of three individuals were frozen one week after the onset of flowering. The greatest delay in the timing of pollination in the 3-year study period was recorded in a quite sunny, but very windy site with lower temperatures in winter and the snow cover persisted longer when compared to the city centre (site V for both, *Alnus* and *Corylus*). At these sites, the synchronization index over the 3-year period ranged from *X* = 0.727 to *X* = 0.96 for hazel and *X* = 0.86 to *X* = 0.976 for alder (Table 4).

**Table 4:** The synchronization index (X) between five sites of *Corylus* and *Alnus* in Szczecin and Rzeszów

Taxon	Station	2009	2010	2011
*Corylus*				
Szczecin	I	0.555	0.455	0.732
	II	0.794	0.256	0.894
	III	0.883	0.715	0.930
	IV	0.711	0.615	0.930
	V	0.727	0.799	0.960
	X mean	0.6045	0.5684	0.8902
Rzeszów	I	0.822	0.521	0.967
	II	0.737	0.833	0.881
	III	0.823	0.866	0.857
	IV	0.657	0.458	0.896
	V	0.443	0.916	0.868
	X mean	0.6964	0.7188	0.8936
*Alnus*				
Szczecin	I	0.764	0.830	0.926
	II	0.561	0.543	0.833
	III	0.638	0.862	0.926
	IV	0.749	0.756	0.900
	V	0.976	0.937	0.860
	X mean	0.7381	0.7861	0.8934
Rzeszów	I	0.589	0.833	0.959
	II	0.740	0.842	0.970
	III	0.700	0.861	0.683
	IV	0.489	0.519	0.969
	V	0.633	0.633	0.677
	VI	0.743	0.712	Site destroyed
	X mean	0.6490	0.7333	0.8516

In Rzeszów, the synchronization of hazel pollination in the 3-year study period also differed. In 2010 and 2011, the studied individuals shed pollen at a similar time. Larger variations in pollination timing were found in 2009 when the value of the synchronization index was *X* = 0.6964. In 2009 and 2011, hazel trees flowered distinctly earlier in the stands located in insolated and wind-sheltered places. In 2009, a clear delay in the timing of hazel pollination was recorded in the stand located at a distance of about 5 km from the pollen trap, in a sunny but very windy place. The synchronization of alder pollination was similar in 2009 and 2010 at all the sites in Rzeszów. In 2011, the average synchronization of pollination was slightly higher. Likewise, in the case of hazel, alder trees flowered distinctly earlier at the sites located in insolated and wind-sheltered places.

### Pollen seasons versus pollination periods

#### Hazel

**Fig. 5:**
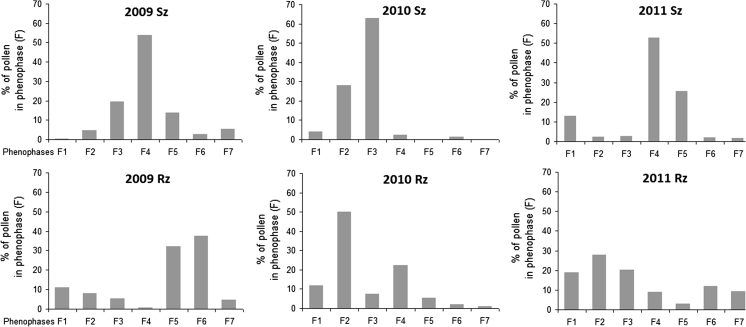
Comparison between phenological and aerobiological data of *Corylus* in Szczecin (Sz) and Rzeszów (Rz)

In Szczecin, the distribution hazel pollen recorded in the successive phenophases varied less than in Rzeszów during 2009–2011. The highest hazel pollen concentrations were found during phenophases of the full pollination period (F3 or F4), with the percentage of pollen grains ranging between 54 and 63 % in these phases (Fig. 5). During the other phases, the numbers of pollen grains ranged from several to >12 %.

In Rzeszów, the full pollination period of hazel (F3–F5) did not coincide with the period of the highest concentrations, and the amounts of pollen recorded during different phenophases varied in each year (Fig. 5). In 2009, the lowest amount of pollen was found in F4, while more than 10 % of the SPI was recorded before the start of the pollination period. The highest amount of pollen (70 %) was found at the end of the full pollination period (F5 and F6). In 2010, the largest amounts of pollen grains were recorded at the beginning of pollination period. As in 2009, more than 10 % of the total seasonal pollen count was recorded before the start of the pollination period. In 2011, more than 65 % of pollen occurred in the first three phases, including as much as 19 % before the onset of the local flowering (Fig. 5). Statistically significant differences were found in the distribution of the numbers of pollen grains in the years of study (*χ* ^2^ test).

#### Alder

**Fig. 6:**
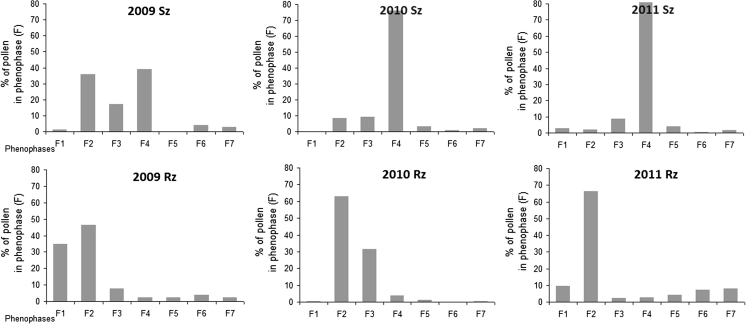
Comparison between phenological and aerobiological data of *Alnus* in Szczecin (Sz) and Rzeszów (Rz)

In Szczecin, in 2010 and 2011, more than 70 % of alder pollen occurred in just one phase (F4). In 2009, the highest amounts of pollen grains were recorded from F2 to F4. Before the onset of pollen shed (F2), only a few percent of the seasonal pollen count was found in the air (Fig. 6). Statistically significant differences were found in the distribution of the numbers of pollen grains during the successive 3 years and also between 2010 and 2011 (*χ* ^2^ test).

In Rzeszów, alder pollen occurred at the highest concentrations at the beginning of pollination, mainly in F2, but as much as 35 % of pollen grains were also found before the beginning of alder pollination in the study area. In 2010, pollen occurred primarily in one phase. More than 60 % of pollen was found in F2, even though this phase only lasted for 5 days. In phase F3, the percentage of pollen grains in the SPI was 31 %. The year 2011 was similar to the two previous years, and the highest amount of pollen was found in F2 (66.3 %; Fig. 6). In spite of these similarities, statistically significant differences were found in the distribution of the numbers (*χ* ^2^ test).

## Discussion

Research carried out in different European countries shows that start dates of the hazel and alder pollen seasons in successive years can vary from 2 weeks to 1.5 months (Piotrowicz and Myszkowska 2006; Emberlin et al. 2007; Smith et al. 2007; Stach et al. 2007; Myszkowska et al. 2010). This can be explained by the influence of meteorological factors, in particular air temperature during the period preceding the pollen season (January–February), which greatly affects the phenology of early flowering species (Frenguelli et al. 1991, 1992; Jato et al. 2004; Rodriguez-Rajo et al. 2004, 2006, Ranta et al. 2008). In addition, Weryszko–Chmielewska et al. (2001) compared the patterns of the pollen seasons in Szczecin, Warsaw, and Lublin and concluded that the geographic location and thereby climatic differences affected the start date of the pollen season. For instance, in Szczecin, the hazel pollen season started about 2–3 weeks earlier and the alder pollen season 1 week earlier than in Lublin, which is located in south-eastern Poland. Myszkowska et al. (2010) also proposed a relationship between the timing of pollen seasons and the geographic location.

The results obtained in our study have not confirmed the thesis of Myszkowska et al. (2010) and Weryszko–Chmielewska et al. (2001) that geographic location influences pollen season characteristics. The results that we obtained varied from year-to-year. In 2009, the pollen seasons of hazel and alder started much later in Szczecin than in Rzeszów, but in 2011, it was the other way around. The season end date showed lower variations and similar tendencies have been reported for other taxa (Wołek and Myszkowska 2008; Myszkowska et al. 2010, Kasprzyk 2011). The results can be also influenced by different ways of reading slides (different counting methods), as has been suggested by Cotos-Yáñez et al. (2013). Similar to Comtois et al. (1999), they have shown that the probability of an estimation error (percentage estimation error) is the higher daily average pollen count. Thus, it can be supposed that the error following from the pollen counts method is lower for alder whose daily average pollen counts and annual total were considerably higher than for hazel pollen.

However, regional differences were found for hazel and alder pollination based on phenological observations of the plant. In Szczecin, the total pollination period and full pollination period started earlier in each year and their duration was longer, which can be a result of the differences in climate type and also the weather pattern. The microclimate of habitats can also be of some significance. Every increase in temperature, even a small one, can initiate pollination of local populations, especially for hazel (Kasprzyk 2010).

Similarities were noted in the curves of the pollen seasons recorded in Szczecin and Rzeszów, in particular the rate that daily average pollen counts increased. This can be attributed to the influence of weather conditions during the period of pollen release. This influence can be seen on both hazel and alder as in most cases the periods of high concentrations of the studied taxa overlap. For example, in Rzeszów, the beginning of February 2009 was warm, and the first hazel and alder pollen grains were already being recorded at that time. This was followed by a severe change in the weather during the second half of February when average daily temperatures dropped below −10 °C. Such weather conditions did not promote intensive and rapid pollen release. On the other hand, such large fluctuations in temperature were not recorded in Szczecin; the number of airborne pollen grains increased along with a distinct increase in temperature, and the pollen season was compact and shorter than in Rzeszów. The local weather conditions influenced the differences in the timing and variation/fluctuation pattern of the pollen seasons. These findings are confirmed in the literature that describes temperature as being the most important factor influencing variations in pollen concentrations (Frenguelli et al. 1991; Myszkowska et al. 2010). In Rzeszów, in 2010, the alder pollen season was very short—only 11 days. The relationship between the weather conditions and pollen concentrations was weaker than expected, but this can be attributed to the shortness of the dataset. A stronger relationship could perhaps be detected over longer periods. The negative correlation between daily average pollen counts and daily mean temperature for alder in Rzeszów was rather surprising because it contradicted our a priori knowledge and should be considered an artifact. The pollen count values show some inert behavior depending not only on the thermal conditions prevailing on the previous days, but also on the number of pollen grains on these days (autocorrelation) (Uruska 2003; Estrella et al. 2006; Stach et al. 2010).

In this study, we have seen that the occurrence of airborne pollen and pollination often coincides, although correlation coefficients are not always significant. These findings have been confirmed by the literature (Latorre 1999; Jato et al. 2007a, b; Kasprzyk and Walanus 2007). It is interesting to note that in both Szczecin and Rzeszów, airborne hazel and alder pollen grains were recorded before or during the start of local pollination, frequently at high concentrations. It has been proposed (e.g. Mimet et al. 2009) that higher minimum air temperatures in urban areas can cause trees to flower earlier, especially in spring, compared to more rural areas. The individuals selected for phenological observations all grew within the area of cities. The pollen traps were placed on the roofs of buildings, and the airborne pollen counts reflect the vegetation and its changes in the region. The pollen season is generally longer than the flowering period, since we take into account all airborne pollen grains, including those originating from local and more distant sources as well as particles that become airborne through re-suspension. Ranta et al. (2006) proved that phenological observations are not sufficient to determine the timing of the main birch pollen season because long-distance transport of pollen may greatly affect the timing of the local birch pollen season. It cannot be excluded that in Rzeszów, alder pollen grains recorded before the flowering period of in situ *A. glutinosa* could have been *A. Incana* pollen grains or *A. glutinosa* pollen grains that were transported over long distances (Mahura et al. 2007; Puc 2007; Kaszewski et al. 2008; Kasprzyk 2010). In microscopic analysis, pollen grains are not distinguished to the level of species and according to the literature, gray alder flowers about 2 weeks earlier (Pancer-Kotejowa and Zarzycki 1980). Though no individuals of this species are found in the flora of the city of Rzeszów, they are often found in the Carpathian Foothills and, theoretically, long-distance transport of pollen from these areas is possible. This is also confirmed by the special location of Szczecin where single *A. incana* individuals have been found at the outskirts of the city (Stachak et al. 2000). For example, in 2007, low alder pollen counts were recorded in Szczecin 1–2 weeks before the main pollen season (Puc 2007). The pollen of the studied taxa, especially hazel, remains in the air in notable concentrations at the end of the pollination period. In spite of the synchronization in pollination witnessed in this study, one should remember that there can be shifts in pollination timing depending on the type of habitat in which these individuals occur (Kasprzyk 2010). These factors are interrelated and depend on the climate, the taxonomic position of the studied taxon, aerodynamic properties of pollen grains, and the location of pollen sources (Latorre 1997, 1999; Fornaciari et al. 2000; Stach et al. 2006; Kasprzyk and Walanus 2007; Jato et al. 2007a, b; Puc et al. 2008).

## Conclusions

We have found a high degree of variation in onset dates of *Corylus* and *Alnus* pollination and pollen seasons of these taxa in the Polish cities of Szczecin and Rzeszów, which could be probably related to the influence of temperature during the period immediately preceding flowering. The occurrence of hazel and alder airborne pollen in the air was significantly influenced by meteorological conditions (*p* < 0.05). No regional differences in the pollen seasons were noted, but the characteristics of pollination were different in the two cities. In Szczecin, pollination started earlier and lasted longer. The synchronization of hazel and alder pollination in Szczecin and Rzeszów varied over the study period. Hazel and alder trees flowered notably earlier in stands located in places that were exposed to sunlight and sheltered from the wind. On the other hand, a delay in the timing of pollination was observed in quite sunny but very windy sites. In Rzeszów, maximum hazel pollen concentrations did not coincide with the period of full pollination (defined as between 25 % hazel and alder and 75 % of flowers open). Conversely, in Szczecin, the highest hazel pollen concentrations were recorded during phenophases of the full pollination period. The period when the highest alder pollen concentrations were recorded varied between sites, with Rzeszów recording the highest concentrations at the beginning of pollination and Szczecin recording alder pollen throughout the full pollination period. Substantial amounts of hazel and alder pollen grains were recorded in the air of Rzeszów (but not Szczecin) before the onset of the respective pollen seasons, which could be probably related to the long-distance transport.

## References

[CR1] Aboulaïch N, Bouziane H, El Kadiri M, Riadi H (2008). Male phenology and pollen production of *Cupressus sempervirens* in Tetouan (Morocco). Grana.

[CR2] APG II System (2003). An update of the angiosperm phylogeny group classification for the orders and families of flowering plants: APG II. Botanical Journal of the Linnean Society.

[CR3] Brzeźniak, E. (2007). Tendencje zmian opadów atmosferycznych w Karpackim Wschodnim regionie opadowym. *Problemy Zagospodarowania Ziem Górskich, 54,* 71–81 [Tendency in precipitation change in East Carpathian rainfall region].

[CR4] Bugała, W. (2000). Drzewa i krzewy. *Państwowe Wydawnictwo Rolnicze i Leśne.* Warszawa (Trees and shrubbery).

[CR5] Cenci CA, Putazalis M, Lorenzetti MC, Lieth H, Schwartz MD (1997). Forecasting an thesis dates of wild vegetation on the basis of thermal and photothermal indices. Phenology in seasonal climates.

[CR6] Comtois P, Alcazar P, Néron D (1999). Pollen counts statistic and its relevance to precision. Aerobiologia.

[CR7] Cotos-Yáñez TR, Rodríguez-Rajo FJ, Pérez-González A, Aira MJ, Jato V (2013). Quality control in aerobiology: Comparison different slide reading methods. Aerobiologia.

[CR8] Črepinšek Z, Kajfež-Bogataj L, Bergant K (2006). Modelling of weather variability effect on fitophenology. Ecological Modelling.

[CR9] D’Amato G, Cecchi L, Bonini S, Nunes C, Annesi-Maesano I, Behrendt H (2007). Allergenic pollen and pollen allergy in Europe. Allergy.

[CR10] Emberlin J, Smith M, Close R, Adams-Groom D (2007). Changes in the pollen season of the early flowering trees *Alnus* spp. and *Corylus* spp. in Worcester United Kingdom 1996–2005. International Journal of Biometeorology.

[CR11] Estrella N, Menzel A, Krämer U, Behrendt H (2006). Integration of flowering dates in phenology and pollen counts in aerobiology: Analysis of their spatial and temporal coherence in Germany (1992–1999). International Journal of Biometeorology.

[CR12] Fornaciari M, Galan C, Mediavilla A, Dominguez E, Romano B (2000). Aeropalynological and phenological study in two different Mediterranean olive areas: Cordoba (Spain) and Perugia (Italy). Plant Biosystems.

[CR13] Frenguelli G, Bricchi E, Romano B, Mincigrucci G, Ferranti F, Antognozzi E (1992). The role of air temperature in determining dormancy release and flowering of *Corylus avellana* L. Aerobiologia.

[CR14] Frenguelli G, Spieksma FTM, Bricchi E, Romano B, Mincigrucci G, Nikkels AH (1991). The influence of air temperature on the starting dates of the pollen season of *Alnus* and *Populus*. Grana.

[CR15] Guardia R, Belmonte J (2004). Phenology and pollen production of *Parietaria judaica* L. in Catalonia (NE Spain). Grana.

[CR16] Hájková L, Nekovář J, Richterová D (2009). Temporal and spatial variability in allergy-triggering phenological phases of hazel and alder in Czechia. Folia Oecologica.

[CR17] Hirst JM (1952). An automatic volumetric spore trap. Annals of Applied Biology.

[CR18] Huntley B, Birks HJB (1983). An atlas of past and present maps for Europe: 0–13000 years ago.

[CR19] Jalas, J., & Suominen, J. (1988). Atlas Florae Europeae. In Distribution of vascular plants in Europe, II (Eds.). Cambridge: Cambridge University Press.

[CR20] Jato V, Méndez J, Rodríguez-Rajo FJ, Dacosta N, Aira MJ (2004). Heat and chill requirements of *Fraxinus* flowering in Galicia (NW Spain). Grana.

[CR21] Jato V, Rodríguez-Rajo FJ, Aira MJ (2007). Use of *Quercus ilex subsp. ballota* phenological and pollen production data for interpreting *Quercus* pollen curves. Aerobiologia.

[CR22] Jato V, Rodríguez-Rajo FJ, Aira MJ (2007). Use of phenological and pollen-production data for interpreting atmospheric birch pollen curves. Annals of Agricultural and Environmental Medicine.

[CR23] Jato V, Rodríguez-Rajo FJ, Alcázar P, De Nuntiis P, Galán C (2006). May the definition of pollen season influence aerobiological results?. Aerobiologia.

[CR24] Kasprzyk I (2010). Początek sezonów pyłkowych olszy i leszczyny a początek pylenia w różnych warunkach siedliskowych Rzeszowa. Alergoprofil.

[CR25] Kasprzyk I (2011). Time-series analysis of pollen seasons in Rzeszów (SE Poland) in 1997–2005 with reference to phenology.

[CR26] Kasprzyk I, Walanus A (2007). Flowering and airborne pollen—a novel statistical approach. Acta Agrobotanica.

[CR27] Kaszewski BM, Pidek IA, Piotrowska K, Weryszko-Chmielewska E (2008). Annual pollen sums of Alnus in Lublin and Roztocze in the years 2001–2007 against selected meteorological parameters. Acta Agrobotanica.

[CR28] Kozłowski TT (1971). Growth and development of trees.

[CR29] Koźmińska, B., & Wojciechowska, D. (2001). Szczecin z daleka i z bliska (Szczecin from a distance and from near by) (pp. 60–79). Zapol: Muzeum Narodowe, Szczecin.

[CR30] Koźmiński C, Czarnecka M, Jasnowska J (1996). Klimat miasta Szczecina i okolicy. Stan Środowiska Miasta i Rejonu Szczecina.

[CR31] Kożuchowski K, Degirmendžić J (2005). Contemporary changes of climate in Poland: Trends and variation in thermal and solar conditions related to plant and vegetation. Polish Journal of Ecology.

[CR32] Latorre F (1997). Comparison between phenological and aerobiological patterns of some arboreal species of Mar del Plata (Argentina). Aerobiologia.

[CR33] Latorre F (1999). Differences between airborne pollen and flowering phenology of urban trees with reference to production, dispersal and interannual climate variability. Aerobiologia.

[CR34] Łukasiewicz A (1984). Potrzeba ujednolicenia metody fenologicznej w polskich ogrodach botanicznych i arboretach. Wiadomości Botaniczne.

[CR35] Mahura AG, Korsholm US, Baklanov AA, Rasmussen A (2007). Elevated birch pollen episodes in Denmark: Contributions from remote sources. Aerobiologia.

[CR36] Mimet A, Pellissier V, Quénol H, Aguejdad R, Dubreuil V, Rozé F (2009). Urbanisation induces early flowering: Evidence from *Platanus acerifolia* and *Prunus cerasus*. International Journal of Biometeorology.

[CR37] Myszkowska D, Jenner B, Puc M, Stach A, Nowak M, Malkiewicz M (2010). Spatial variations in dynamics of *Alnus* and *Corylus* pollen season in Poland. Aerobiologia.

[CR38] Niedźwiedź, T. (1981). Sytuacje synoptyczne i ich wpływ na zróżnicowanie przestrzenne wybranych elementów klimatu w dorzeczu górnej Wisły. *Rozprawy Habilitacyjne nr 58. Uniwersytet Jagielloński, Kraków* (Synoptic situations and their influence on spatial differentiation of chosen elements of climate of the upper Vistula a Basin).

[CR39] Niedźwiedź, T. (2004). Kalendarz sytuacji synoptycznych dla dorzecza górnej Wisły (1997–2004); plik komputerowy dostępny w *Katedrze Klimatologii, Wydział Nauk o Ziemi Uniwersytetu Śląskiego, Sosnowiec (*Calendar of synoptic situations for Upper Wisła Basin; computer packet available in Climatology Department, Earth Science Faculty, Śląsk University).

[CR40] Nilsson S, Persson S (1981). Tree pollen spectra in the Stockholm region (Sweden) 1973–1980. Grana.

[CR41] Ollerton J, Lack A (1998). Relationships between flowering phenology, plant size and reproductive success in *Lotus corniculatus* (Fabaceae). Plant Ecology.

[CR42] Pancer-Kotejowa, E., & Zarzycki, K. (1980). Zarys ekologii (Outline of ecology). In: S. Białobok (Ed.), *Olsze Alnus Mill. (Alders)* (pp. 229–257). Warsaw: Instytut Dendrologii PAN.

[CR43] Piotrowicz K, Myszkowska D (2006). Początek sezonów pyłkowych leszczyny na tle zmienności klimatu Krakowa. Alergologia, Immunologia.

[CR44] Puc M (2003). Characteristic of pollen allergens. Annals of Agricultural and Environmental Medicine.

[CR45] Puc M (2003). Pyłek wybranych taksonów drzew w powietrzu Szczecina w latach 2000–2002. Annales Universitatis Mariae Curie-Skłodowska.

[CR46] Puc M (2007). The effect of meteorological conditions on hazel and alder pollen concentration in the air of Szczecin. Acta Agrobotanica.

[CR47] Puc M (2012). Artificial neural network model of the relationship between Betula pollen and meteorological factors in Szczecin (Poland). International Journal of Biometeorology.

[CR48] Puc M, Grinn-Gofroń A, Myśliwy M, Wolski T, Rapiejko P, Sieczka J (2008). Okres kwitnienia brzozy w 2008 roku w Szczecinie a zagrożenie alergenami pyłku tego drzewa. Alergoprofil.

[CR49] Ranta H, Hokkanen T, Linkosalo T, Laukkanen L, Bondestam K, Oksanen A (2008). Male flowering of birch: Spatial synchronization, year-to-year variation and relation of catkin numbers and airborne pollen counts. Forest Ecology and Management.

[CR50] Ranta H, Kubin E, Siljamo P, Sofiev M, Linkosalo T, Oksanen A (2006). Long distance pollen transport cause problem for determining the timing of birch pollen season in Fennoscandia by using phenological observations. Grana.

[CR51] Rapiejko P, Lipiec A, Wojdas A, Jurkiewicz D (2004). Threshold pollen concentration necessary to evoke allergic symptoms. International Review of Allergology and Clinical Immunology.

[CR52] Rodriguez-Rajo FJ, Dopazo A, Jato V (2004). Environmental factors affecting the start of pollen season and concentration of airborne *Alnus* pollen in two localities of Galicia (NW Spain). Annals of Agricultural and Environmental Medicine.

[CR53] Rodriguez-Rajo FJ, Valencia-Barrea RM, Vega-Maray AM, Suárez FJ, Fernández-Gonzales D, Jato V (2006). Prediction of airborne *Alnus* pollen concentration by using ARIMA models. Annals of Agricultural and Environmental Medicine.

[CR54] Senata W (1991). Dendrologia 1.

[CR55] Smith M, Emberlin J, Stach A, Czarnecka–Operacz M, Jenerowicz D, Silny W (2007). Regional importance of Alnus pollen as an aeroallergen: A comparative study of *Alnus* pollen counts from Worcester (UK) and Poznań (Poland). Annals of Agricultural and Environmental Medicine.

[CR56] Sokołowska J (1962). Izofeny kwitnienia leszczyny (*Corylus avellana* L.). Rocznik Dendrologiczny.

[CR57] Stach A, Garcia-Mozo H, Prieto-Baena JC, Czarnecka-Operacz M, Jenerowicz D, Silny W (2007). Prevalence of artemisia species pollinosis in western Poland. Impact of climate change on aerobiological trends, 1995–2004. Journal of Investigational Allergology and Clinical Immunology.

[CR58] Stach, A., Kasprzyk, I., Puc, M., Weryszko-Chmielewska, E., Piotrowska, K., Stach, A. et al. (2010).Temporal and space-time autocorrelation of daily concentrations of *Alnus,**Betula* and *Corylus* pollen in Poland. Abstract 9th ICA (International Congress of Aerobiology, 23–27 08 2010, Buenos Aires, Argentina).

[CR59] Stach A, Kluza-Wieloch M, Zientarska A (2006). The phenology of flowering and fluctuations of airborne pollen concentrations of selected trees in Poznań, 2003–2004. Acta Agrobotanica.

[CR60] Stachak, A., Grinn, U., Haas-Nogal, M., Kubus, M., Nowak, G., & Nowakowska, M. (2000). *Zieleń Szczecina*. (pp. 64–273). Szczecin: Oficyna In Plus Press (Greenery of Szczecin).

[CR61] StatSoft Inc (2008). STATISTICA (data analysis software system), version 9.0 www.statsoft.com.

[CR62] Sugita S, Hicks S, Sormunen H (2010). Absolute pollen productivity and pollen-vegetation relationships in northern Finland. Journal of Quaternacy Science.

[CR63] Suszka, B. (1980). Rozmnażanie generatywne. In: S. Białobok (Ed.) Olsze *Alnus* Mill (pp. 99–144). Warszawa–Poznań: PWN.

[CR64] Święs, F. (1993). *Roślinność synantropijna miasta Rzeszów*. Wyd. Uniw. Marii Curie-Skłodowska, Lublin. (The synanthropic vegetation of the city of Rzeszów).

[CR65] Uruska A (2003). Wpływ wybranych czynników meteorologicznych na zmianę koncentracji ziaren pyłku drzew w atmosferze Gdańska. Ann. UMCS, Sec. EEE, Horticultura.

[CR66] Weryszko–Chmielewska E, Puc M, Rapiejko P (2001). Comparative analysis of pollen counts of *Corylus*, *Alnus* and *Betula* in Szczecin, Warsaw and Lublin (2000–2001). Annals of Agricultural and Environmental Medicine.

[CR67] Weryszko-Chmielewska, E., & Rapiejko, P. (2007). Analysis of *Alnus* spp. pollen seasons in Lublin and Warszawa (Poland), 2001–2007. *Acta Agrobotanica, 60*(2), 87–97.

[CR68] Wołek J, Myszkowska D (2008). Strategia badań aerobiologicznych. Alergologia and Immunologia.

[CR69] Woś A (1999). Klimat Polski.

[CR70] Zając A*., &* Zając M. (Eds.) (2001). Distribution atlas of vascular plants in Poland. Laboratory of Computer Chorology, Institute of Botany, Jagiellonian University, Krakow, ss. 715.

[CR71] Ziernicka-Wojtaszek A, Zawora T (2008). Regionalizacja termiczno-opadowa Polski w okresie globalnego ocieplenia. Acta Agrophysica..

